# Neurofilament light chain and S100B serum levels are associated with disease severity and outcome in patients with aneurysmal subarachnoid hemorrhage

**DOI:** 10.3389/fneur.2022.956043

**Published:** 2022-08-03

**Authors:** Zhangming Zhou, Junyi Zeng, Shui Yu, Ying Zhao, Xiaoyi Yang, Yiren Zhou, Qingle Liang

**Affiliations:** ^1^Department of Neurosurgery, Dujiangyan Medical Center, Chengdu, China; ^2^Department of Clinical Laboratory, Taihe Hospital, Hubei University of Medicine, Shiyan, China; ^3^Department of Medicine, First Clinical School, Guangzhou Medical University, Guangzhou, China; ^4^Department of Neurology, Chengdu 3rd People's Hospital, Chengdu, China; ^5^Department of Clinical Laboratory Medicine, First Affiliated Hospital, Third Military Medical University (Army Medical University), Chongqing, China

**Keywords:** aneurysmal subarachnoid hemorrhage, neurofilament protein light, S100B, biomarkers, prognosis

## Abstract

**Objectives:**

Serum neurofilament light chain (NfL) is a biomarker for neuroaxonal damage, and S100B is a blood marker for cerebral damage. In the present study, we investigated the relationship between serum NfL and S100B levels, severity, and outcomes in patients with aneurysmal subarachnoid hemorrhage (aSAH).

**Methods:**

We prospectively recruited aSAH patients and healthy controls between January 2016 and January 2021. Clinical results included mortality and poor outcomes (modified Rankin scale score of 3-6) after 6 months. The ultrasensitive Simoa technique was used to evaluate NfL levels in the blood, and ELISA was used to detect S100B.

**Results:**

A total of 91 patients and 25 healthy controls were included in the study, with a death rate of 15.4%. The group of aSAH patients had significantly higher serum levels of NfL and S100B (*P* < 0.01). Furthermore, the levels of NfL and S100B increased when the Hunt-Hess, World Federation of Neurological Surgeons (WFNS), and Fisher grades increased (*P* < 0.01). Serum NfL and S100B levels were linked to poor prognoses and low survival rates. The blood levels of NfL and S100B were found to be an independent predictor related to 6-month mortality in multivariable analysis. Additionally, the areas under the curves for NfL and S100B levels in serum were 0.959 and 0.912, respectively; the clinical diagnostic critical thresholds were 14.275 and 26.54 pg/ml, respectively; sensitivities were 0.947 and 0.921, and specificities were 0.849 and 0.811.

**Conclusions:**

The NfL and S100B values for aSAH patients within 12 days of admission were considerably associated with Hunt-Hess grade, WFNS, and Fisher grade. The higher the grade, the higher the NfL and S100B value, and the poorer the prognosis. Serum NfL and S100B values could be feasible biomarkers to predict the clinical prognosis of patients with aSAH.

## Introduction

Aneurysmal subarachnoid hemorrhage (aSAH) is the deadliest form of a devastating disease, and poor-grade patients have poorly predicted outcomes. It has been reported that aSAH accounts for approximately 15% of cerebrovascular disease (1, 2). Without effective treatment, the mortality rate of patients with Fisher grade 3, and Hunt-Hess grading scales III or above is as high as 44% (3, 4). The prognosis of aSAH patients is even worse in most low- and middle-income countries owing to the lack of techniques and facilities for craniotomy and interventional embolization. Moreover, aSAH is usually followed by cerebral vasospasm (CVS) or even cerebral infarction in some extreme cases (5, 6). Thus, the early diagnosis and treatment of the disease are imperative, and it is also necessary to judge the prognosis of patients. Nearly 1 year after patients' admission to the hospital, brain damage and subsequent pathological changes occur, especially the long-term damage mechanism of hemoglobin and inflammatory molecules to the brain, fueling increased research interest (7, 8). The basic concept of early brain injury (EBI) is fundamental because it represents that the initial clinical presentation is the most important predictor of outcome (9). Therefore, the Hunt and Hess (H-H) grade, Fisher grades, and the World Federation of Neurological Surgeons (WFNS) scale are commonly used for predicting the prognosis of aSAH. Although it appears important for the outcome, the mechanisms behind brain injury are multifactorial and remain incompletely understood (10). In addition, the clinical predictive scoring system is somewhat subjective, and the patient may be in a state of sedation or coma when admitted to the hospital. In this condition, different doctors may give variable scores. Furthermore, WFNS only explained a minor proportion of variance in the outcome, and the contribution of the other predictors was substantially lower. To better understand the brain damage and the mechanism caused by aSAH, it is necessary to identify reliable biomarkers to promote further research and future clinical implementation.

Neurofilaments have three subunits; neurofilament light protein (NfL) is the smallest, neuron-specific protein abundant in myelinated axons and released into the extracellular compartment when neuronal damage occurs (11). Recent studies have proved cerebrospinal fluid (CSF) or plasma NfL as a useful neuroaxonal impairment biomarker in a diverse range of neurological diseases including degenerative conditions (11–13), as well as cerebral hemorrhage (14) and traumatic (15) brain injuries. A few studies have shown that the level of NfL in the plasma of patients with aSAH continues to increase, and the level of NfL is related to the severity of the patients' condition when admitted to the hospital as well as long-term outcomes (10, 16). However, most previous researchers focused on the late phase (>72 h) after bleeding, and the association among blood-borne neurofilament levels in the early brain injury phase, disease severity on admission to hospital, and the long-term consequences has not been previously explored.

S100B protein can effectively reflect brain tissue damage (17). In subsequent studies, many authors related pathologically increased serum levels of the S100B with head trauma (18). S100 B protein is recommended in current traumatic brain injury guidelines and is used in the clinical emergency routine for patients with head trauma. Moreover, S100B has been a well-studied marker for ischemic injury, stroke, coronary artery bypass graft surgery, and aSAH (19, 20). NfL and S100B release characteristics in patients with aSAH have not been reported.

In the present study, the levels of NfL and S100B in patients with aSAH were detected, and the correlation between them was also examined to explore the pathogenesis of aSAH to provide new insights into the clinical diagnosis of aSAH. The increase in serum NfL and S100B concentration following surgery or medical treatment may reflect disease severity and outcome in patients with aSAH.

## Materials and methods

### Patients and exclusion criteria

A total of 91 patients with aSAH who were diagnosed by computerized tomography (CT) and digital subtraction angiography (DSA) in the Department of Neurosurgery, Dujiangyan Medical Center, Chengdu, China, from January 2016 to January 2021 were enrolled as the observation group. These patients included 37 men and 54 women aged 41–75 years, with an average age of 56.06 ± 6.0 years. The CT scans and DSA images were evaluated by a neuroradiology consultant and scored according to the Fisher grade. Exclusion criteria were a preexisting hemorrhagic disease, treatment with antithrombotic drugs, active cancer or chemotherapy in the previous 3 months, and liver cirrhosis. In addition, patients who had experienced ischemic or hemorrhagic cerebral infarction within the previous 3 months, structural causes of SAH (arteriovenous malformation, tumor, or trauma), and autoimmune diseases were excluded. Twenty-five healthy people who matched the age and sex of physical examination in our hospital in the same period were selected as the health group. This study was discussed and approved by the hospital ethics committee. Written informed consent for participation was not required for this study in accordance with the national legislation and the institutional requirements.

### Blood collection

All routine examinations were conducted immediately after admission to the observation group. Blood samples were collected on day 0, day 1, day 3, and day 10–12 after hemorrhage. Immediately, samples were centrifuged at 3,000 g for 10 min at 4°C and then stored at −80 °C until analysis. At the same time, serum NfL and S100B levels in the control group were examined.

### Serum neurofilament light assay

The serum NfL levels were detected by a commercial nuclear factor kit (Quanterix, Lexington, MA, USA) of single molecular array immunoassay (SIMOA) on an HD-1 analyzer (Quanterix) (21). Briefly, samples were thawed at 25 °C, and vortexed, 10,000 RCF centrifugation was applied for 5 min. The samples were diluted with sample diluent at a ratio of 1:4 and bonded to paramagnetic magnetic beads on the instrument, which were coated with human NfL-specific antibodies. Then the biotinylated anti-NfL detection antibody was conjugated to streptavidin-β-galactosidase complex, and fluorescence detection was performed. Sample concentrations were calculated from a standard curve, fitted using a four-parameter logistic curve.

### Serum S100B assay

An enzyme-linked immunosorbent assay kit detected the serum S100B level (purchased from Elabscience Company, Wuhan, China). Briefly, samples were thawed at 25 °C, vortexed, and 1750 RCF centrifugation was applied for 5 min. The samples were diluted with sample diluent at a ratio of 1:2 and added to microplate wells, which were coated with human S100B-specific antibodies. Then the biotinylated anti-S100B detection antibody was incubated with samples. Sample concentrations were calculated from a standard curve, fitted using a four-parameter logistic curve.

### Clinical scales

**Figure 1:**
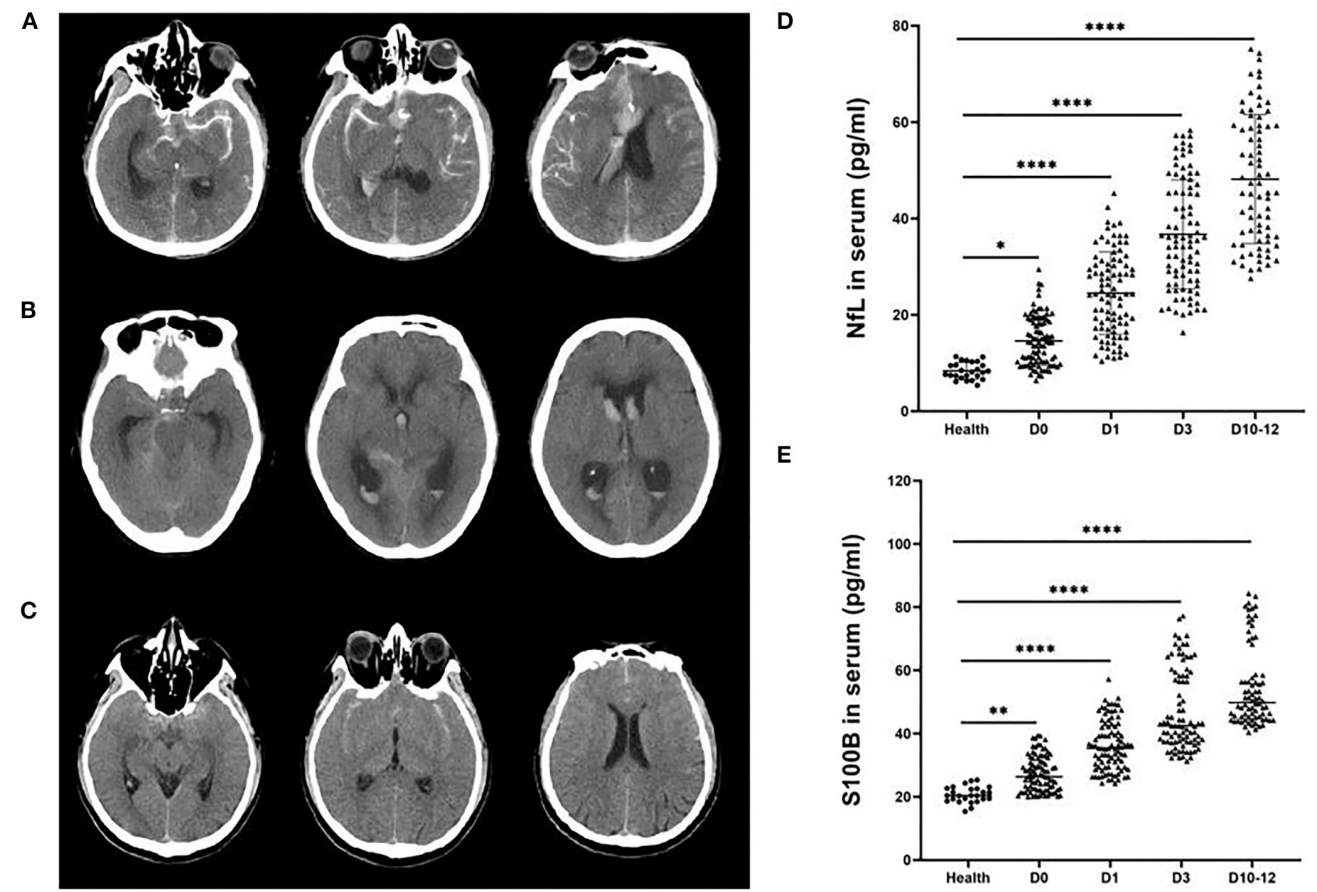
Representative head computed tomography (CT) scan of patients with aneurysmal subarachnoid hemorrhage (aSAH). **(A)** The head CT scans of a patient with a Hunt-Hess grade IV aSAH with global and focal edema. **(B)** The head CT scans of an aSAH patient with a Hunt-Hess grade III with hydrocephalus. **(C)** The head CT scans of an aSAH patient with a Hunt-Hess grade II without edema or hydrocephalus. NfL **(D)** and S100B **(E)** levels were significantly elevated in the serum of aSAH patients compared with control patients at each period. Ultrasensitive Simoa and ELISA results showed significant differences between the health group and patients with different Hunt-Hess grades. ***P < 0.001.

CT images were used to grade the observation group. Fisher grading standard: grade 1: no blood was found in the subarachnoid space; grade 2: a thin layer of blood in the scanning layers such as longitudinal fissure and insular cistern, thickness < 1 mm, or blood diffusely distributed in the subarachnoid space; grade 3: localized blood clot in the subarachnoid area, or blood clot thickness ≥ 1 mm in vertical layers grade 4: blood clot in the brain or ventricle, no or with diffuse subarachnoid hemorrhage (Figures 1A–C). The correlation between Fisher grade, serum NfL, and S100B levels after hemorrhage in the observation group was analyzed.

Patients were followed-up for 6 months after aSAH. The primary outcome was a functional state. Moreover, we assessed functional status using the modified Rankin scale (mRS) scores. Good outcome was defined as having the ability to perform activities of daily living (mRS score ≤ 2). Poor outcome was defined as an mRS score ≥ 3. According to the survival and mRS a half-year later, the patients in the observation group were divided into survival, non-survival, good prognosis, and poor prognosis subgroups. The serum NfL levels of different subgroups were compared, and the correlation between the prognosis and serum NfL level in the observation group was analyzed. The serum NfL level ROC curve predicting the prognosis of aneurysmal subarachnoid hemorrhage was evaluated.

### Statistical analysis

SPSS 25.0 software was used to process the data. The measurement data were expressed as (mean ± SEM), and a one-way ANOVA was used to compare groups. The counting data were described by rate. Furthermore, the receiver operating curve (ROC) was used to study the value of serum NfL, S100B, in evaluating the poor prognosis of aSAH. Spearman correlation analysis was used to analyze the correlation among serum NfL, S100B and CT grade, Hunt-Hess grade, WFNS score, and Fisher grade. The logistic regression model was used to explore whether serum NfL and serum S100B were independent risk factors of aSAH.

## Results

Ninety-one subjects were enrolled in this study, among whom 53 were in a good outcome group (mRS scores 0–2) and 38 were in a poor outcome group (mRS scores 3–6). The poor outcome group had more patients with an III-V Hunt-Hess grade than the good outcome group (*P* < 0.01). The same results were observed in the WFNS (*P* < 0.01) and Fisher (*P* < 0.01) grades and higher frequency of hydrocephalus (*P* < 0.01). The major aneurysm sites were the anterior communicating artery and internal carotid artery (32.97 and 39.56%, respectively) (Table 1).

**Table 1:** Baseline demographics and clinical findings of patients with aneurysmal subarachnoid hemorrhage.

	**Overall**	**Good**	**Poor**	
		**outcome**	**outcome**	
**Variables**	**(*n* = 91)**	**mRS (0–2)**	**mRS (3–6)**	***P* value**
		**(*n* = 53)**	**(*n* = 38)**	
Age (years)*		53.49 ± 4.26	59.65 ± 6.15	<0.01
**Gender**, ***n*** **(%)**				
**Female/male**	54/37	33/20	19/19	
**Clinical findings**, ***n*** **(%)**				
H-H grade				
I-II	47 (51.65%)	44	3	<0.01
III-V	44 (48.35%)	9	35	
WFNS score				
Good (I–III)	61 (67.03%)	53	8	<0.01
Poor (IV–V)	30 (32.97%)	0	30	
Fisher Grade, *n* (%)				
2	35 (38.46%)	33	2	<0.01
3	40 (43.96%)	20	20	
4	16 (17.58%)	0	16	<0.01
Brain edema, *n* (%)	31 (34.07%)	16	15	
Hydrocephalus, *n* (%)	29 (31.90%)	9	17	<0.01
CVS, n (%)	38(41.80%)	9	29	<0.01
**Aneurysm site**, ***n*** **(%)**	89			
ACoA	30 (32.97%)	22	8	
ICA	36 (39.56%)	30	6	
ACA	2 (2.20)	1	1	
MCA	15 (16.50)	9	6	
Vert.A	2 (2.20)	1	1	
PICA	1 (1.10)	1	0	
BA	2 (2.20)	1	1	
SCA	1 (1.10)	1	0	
PCA	2 (2.20)	1	1	

* Mean ± SD, aSAH, aneurysmal subarachnoid hemorrhage, ACA, anterior cerebral artery, ACoA, anterior communicating artery, ICA, internal carotid artery, mRS, modified Rankins Score, MCA, middle cerebral artery, PCoA, posterior communicating artery, PICA, posterior inferior cerebellar artery, SCA, superior cerebellar artery, Vert.A, vertebral artery, BA, basilar artery, PCA, posterior cerebral artery, H-H grade, Hunt-Hess grade, CVS, cerebral vasospasm, WFNS score, World Federation of Neurological Surgeons, WBC, white blood cells, APTT, activated partial thromboplastin time, INR, International Normalized Ratio.

The levels of NfL and S100B in serum of patients with aSAH were measured in different various periods (days 0, 1, 3, and 10–12, the day of aSAH patient admission was recorded as day 0). The levels of NfL (Figure 1D) and S100B (Figure 1E) were substantially increased in the serum of patients with aSAH in different periods compared with the health group (*P* < 0.001).

**Figure 2:**
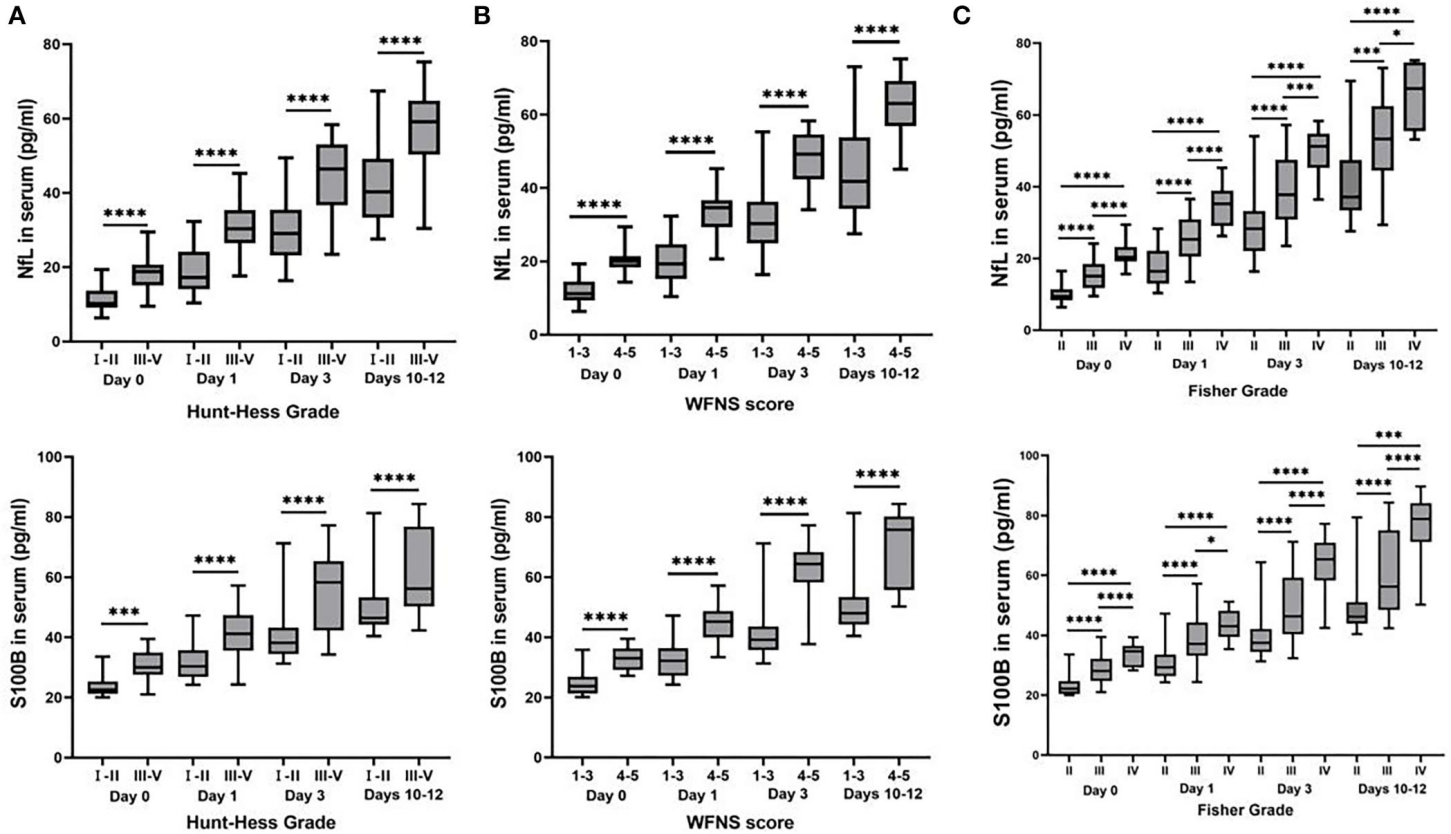
Box plots of concentrations of inflammatory cytokines classified by Hunt-Hess grade, World Federation of Neurological Surgeons (WFNS) grade, and Fisher grade. The serum NfL and S100B levels in patients with aSAH were considerably different from those various evaluations of neurological diseases. **(A)** The higher levels of serum NfL and S100B were detected in aSAH patients with H-H grades III and IV, compared with gradesIand II at each time point (*P* < 0.01). **(B)** Serum NfL and S100B levels were much higher in aSAH patients with WFNS scores of 4 and 5 than those with WFNS scores of 1–3 at each time point (*P* < 0.0001). **(C)** The serum NfL and S100B concentrations in aSAH patients differed significantly in various Fisher grades at each time point (*P* < 0.05). All data were presented as mean ± SEM. *P < 0.05; **P < 0.01; ***P < 0.001; ****P < 0.0001.

We found an increasing trend of NfL and S100B in aSAH patients with higher (H-H grades), World Federation of Neurological Surgeons grades (WFNS grades), and Fisher grades within days 0, 1, 3, and 10–12 (Figure 2). Compared with the lower H-H grades (grades I and II), the levels of NfL, and S100B in higher H-H grades (grades III to IV; *P* < 0.0001) were significantly higher (Figure 2A). The levels of NfL and S100B were considerably higher in aSAH patients with high WFNS grades (grades IV and V; *P* < 0.0001) (Figure 2B). Additionally, the same results were found in aSAH patients with different Fisher grades, The higher the serum NfL and S100B levels, the higher the corresponding Fisher grade, and there was a significant difference among grades II, III, and IV (*P* < 0.001). Meanwhile, the statistics results indicated that the correlation between NfL and Fisher Grade was stronger than that of S100B. The R squared value of S100B was all <0.5 on different days, but NfL was more than 0.5 except on days 10–12, the definition of R squared <0.5 was uncorrelated (Figure 2C).

**Figure 3:**
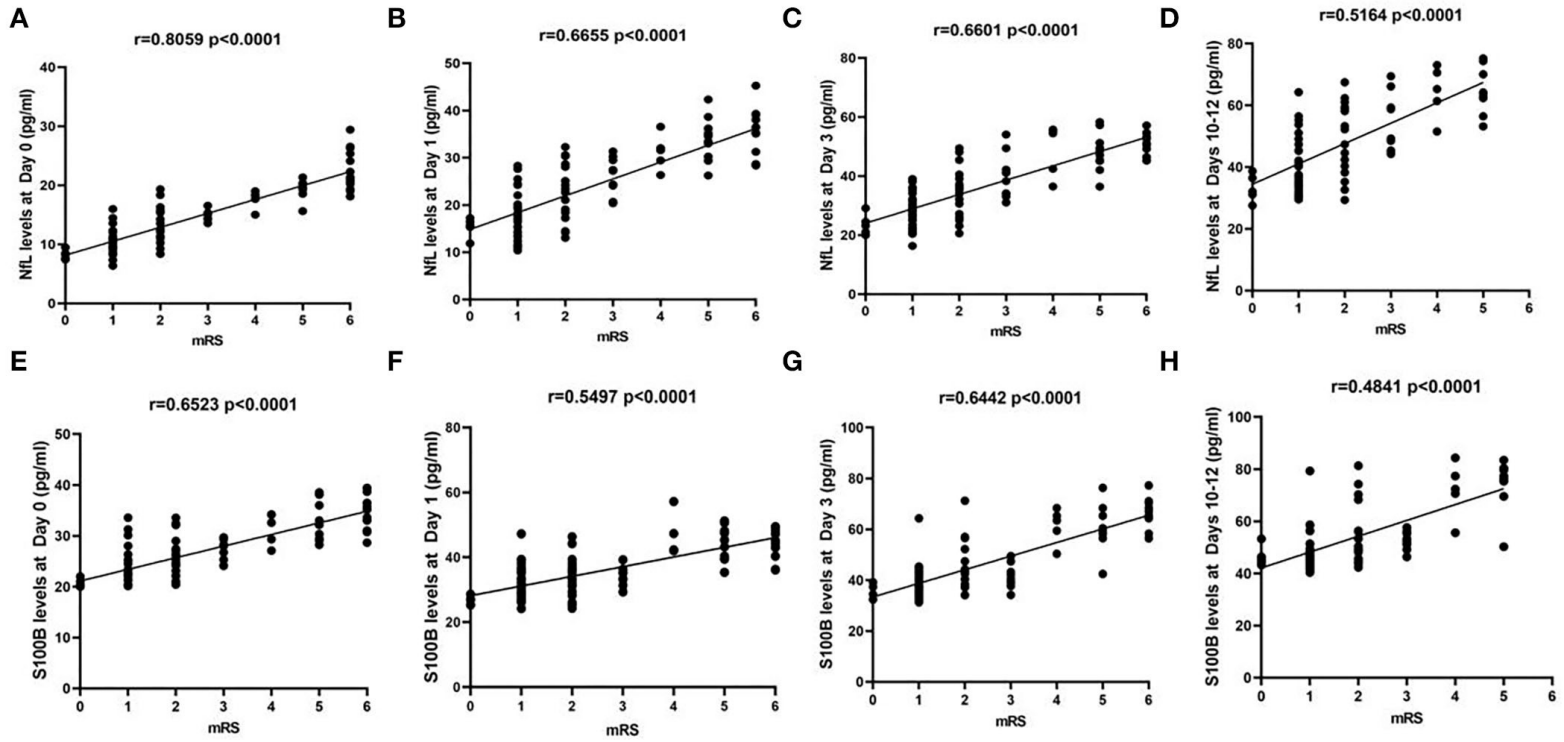
**(A–D)** The levels of aSAH patients serum NfL (Day 0, *r* = 0.8059, Day 1, *r* = 0.6550, Day 3, *r* = 0.5960, and Days 10–12, *r* = 0.6468, respectively; *P* < 0.0001), **(E–H)** S100B (Day 0, *r* = 0.6530, Day 1, *r* = 0.5554, Day 3, *r* = 0.6200, and Days 10–12, *r* = 0.6345, respectively; *P* < 0.0001) were significantly correlated with modified Rankin scale scores at each time points.

**Figure 4:**
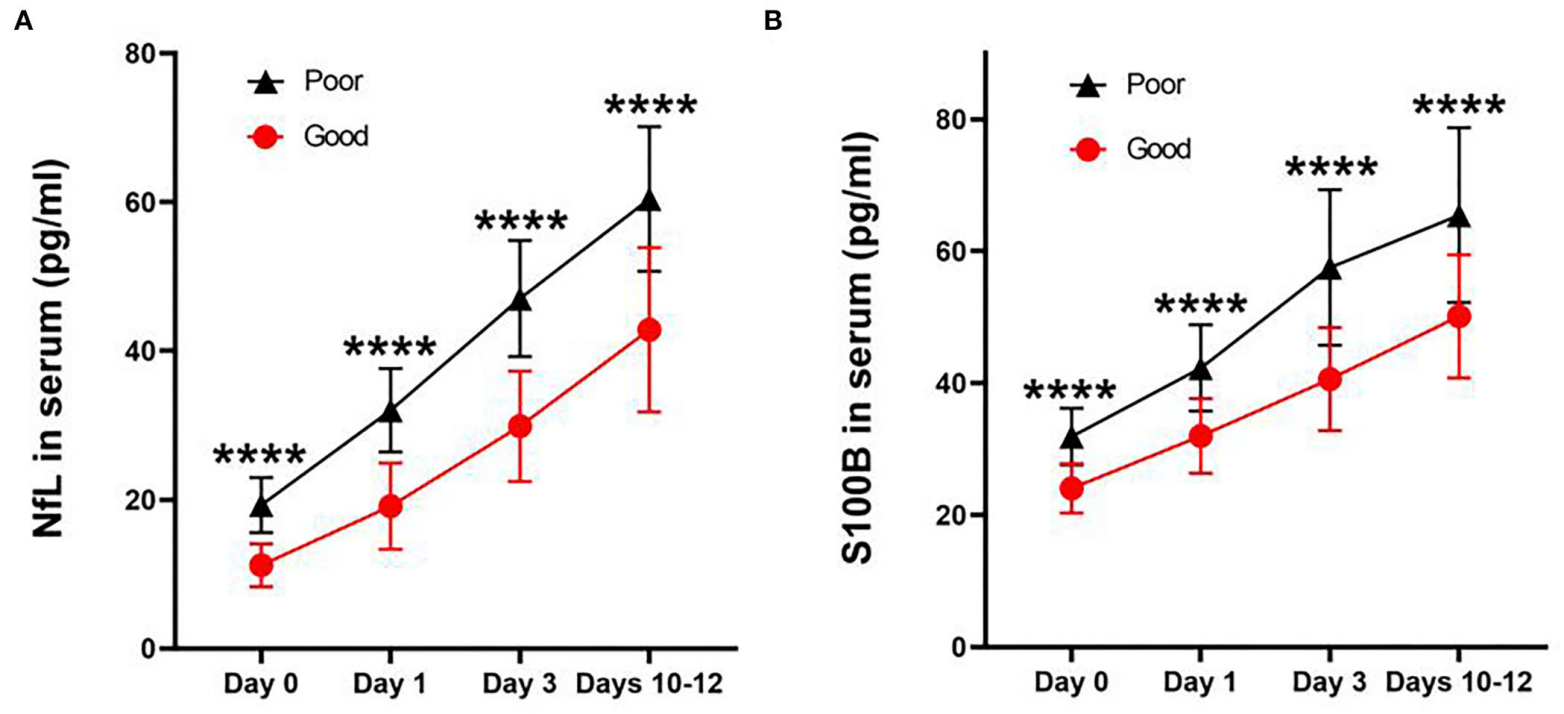
Line charts of concentration of NfL **(A)** and S100B **(B)** in serum after aneurysmal subarachnoid hemorrhage at each time point. Both concentrations were significantly higher in those with poor outcomes than in good outcomes (*P* < 0.0001). All data were presented as mean ± SEM. ^****^P < 0.0001.

For all subjects, we defined good and poor outcomes for patients with an mRS score of ≤2 and ≥3 at 6 months, respectively. We found that the levels at each time point were significantly correlated with poor outcomes when correlating NfL (Figures 3A–D) and S100B (Figures 3E–H) serum levels in aSAH patients with mRS scores (Figure 3). In addition, aSAH patients with a poor outcome had meaningfully higher serum NfL (Figure 4A) and S100B (Figure 4B) levels on days 0, 1, 3, and 10–12 than those with a good outcome.

**Figure 5:**
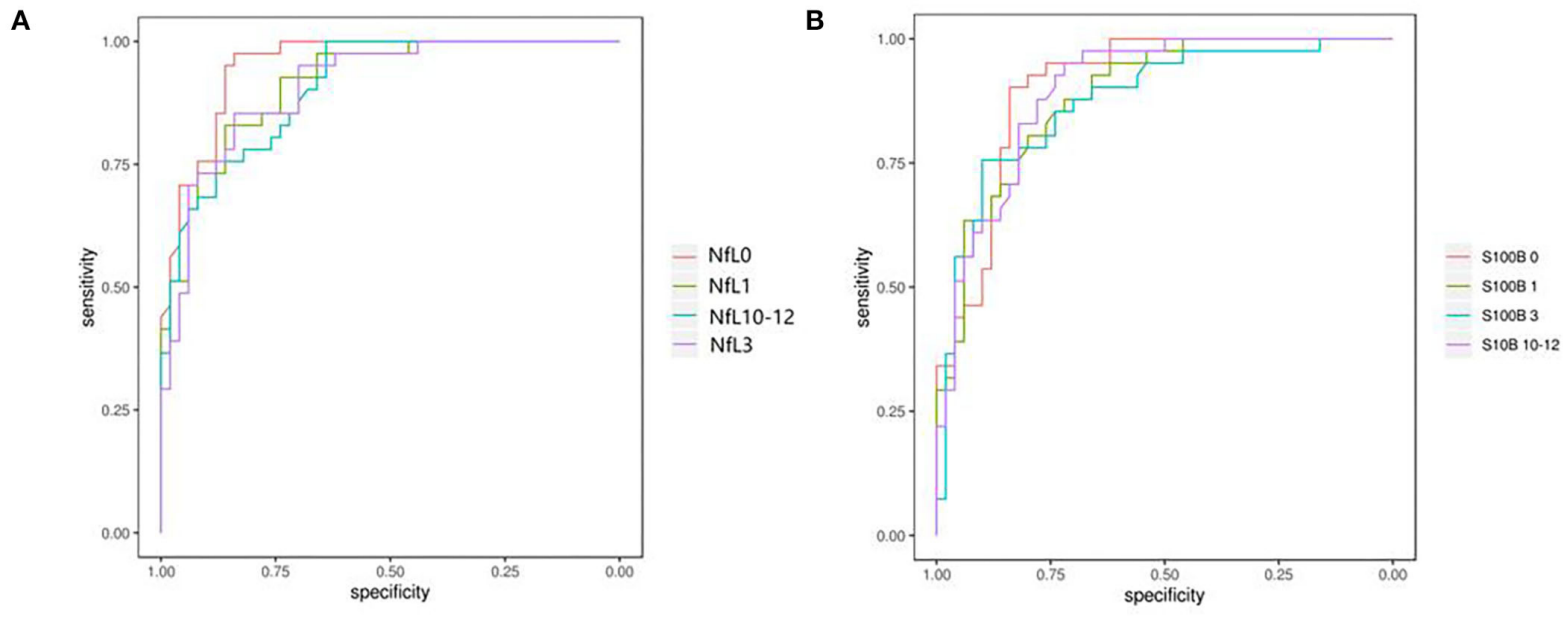
**(A,B)** Receiver operating characteristics revealed a sensitivity/specificity of NfL and S100B. The areas under curves for the levels of NfL and S100B in the serum were 0.959 and 0.912, respectively; the clinical diagnostic critical points were 14.275 and 26.54 pg/ml, respectively; sensibilities were 0.974 and 0.921, respectively, and specificities were 0.849 and 0.811, respectively.

Receiver operating curve analysis of serum NfL and S100B levels were applied to predict the prognosis of aSAH. On the day of admission, the AUC of serum NfL and S100B for predicting aSAH was 0.959 and 0.912, respectively. The best cutoff value of serum NfL for predicting the severity of aSAH on the day of admission was 14.275 pg/ml, and the sensitivity and specificity were 0.974 and 0.849, respectively (Figure 5A). The best cutoff value of serum S100B for predicting the survival of aSAH was 26.54 pg/ml, and the sensitivity and specificity were 0.921 and 0.811, respectively (Figure 5B).

## Discussion

In recent years, many researchers have found a variety of biomarkers related to aSAH. These markers play a specific role in the development and prognosis of SAH, but the specificity and sensitivity were not insufficient, and there were few prognostic markers for aSAH (16). Axonal white matter injury caused by hemorrhage was the main factor causing craniocerebral injury. Moreover, NfL was heavily expressed in axonal white matter, which was an essential part of the cytoskeleton and maintained the normal physiological function of axons, including the branching and growth of dendrites. Studies have shown that the level of NfL in CSF of patients with SAH was considerably higher, which can be used to evaluate the condition and prognosis of patients (22). However, because of the invasion of lumbar puncture, routine repeated lumbar puncture was not feasible in the clinic. So far, there is no study for serum NfL levels 3–12 days after admission. Therefore, we used the single molecular array immunoassay (SIMOA) on an HD-1 analyzer method to measure serum NfL levels in patients with aSAH to explore the relationship between NfL and prognosis. This was a highly sensitive immunoassay technology with dedicated hardware and software that quantified analyte concentrations by cingulated capture and reading of immunocomplexes on microbeads. The assay was at least 125 times more sensitive than conventional ELISA while maintaining high analytical performance (21).

Furthermore, the serum NfL levels of 91 patients with aneurysmal subarachnoid hemorrhage and 25 healthy subjects were analyzed. The results showed that the level of serum NfL in the observation group was significantly higher than in the health group. NfL should be detected in the serum of patients with aSAH as soon as admission. The concentration was considerably higher than that in the health group and increased rapidly from admission to days 10–12. This may be related to increased intracranial pressure and hypoxemia after aSAH. Further studies showed that the serum NfL concentration in patients in a poor prognosis subgroup was much higher than that in a good prognosis subgroup, indicating that the level of serum NfL in patients with aSAH was substantially correlated with the risk of severe injury. This was owing to the high expression of NfL in the axon. After being subjected to external forces, the neuron is damaged, and NfL enters the extracellular fluid and blood through the blood-brain barrier until the balance is reached. Therefore, the serum NfL concentration in patients with more severe aSAH was higher than in patients with mild aSAH.

S100B protein belongs to the S100 family and mainly exists in glial cells. Moreover, S100B is related to tumors, mental disorders, epilepsy, and brain injury. Under normal circumstances, S100B protein cannot pass through the blood-brain barrier, but aSAH leads to brain tissue damage, and the destruction of brain cells and the blood-brain barrier cause the blood S100B to increase rapidly, which has a specific value for the diagnosis and treatment of brain injury (23). The results of this study also demonstrate that the serum S100B protein increases quickly after an acute brain injury caused by aSAH, which is considerably higher than the health group, and the levels of serum S100B rise over time.

Further analysis showed less correlation between S100B protein and CT grade of aSAH, but serum NfL was positively correlated with CT grade of aSAH, the sensitivity of the detection methods may account for this difference. we used the SIMOA method to detect NfL while S100B was ELISA, the former is much more sensitive than the latter. In addition, S100B was mainly used to indicate brain injury, the volume, and area of local injury of brain injury were larger than that of aSAH. Compared with NfL, the distribution of S100B was found to be higher in gray matter and lower in white matter in the cerebral cortex. The specific molecular mechanism needs to be further studied. For aSAH, local white matter damage may be more, resulting in a greater correlation between NfL and prognosis, which may be a better predictor compared with S100B. Demonstrating the degree of aSAH, NfL had some advantages compared with S100B protein.

In this study, by drawing the ROC curve, we found that the AUC of serum NfL and S100B on the day of admission to predict aSAH was 0.959 and 0.912, respectively. The sensitivity and specificity of predicting the risk in the severity of patients were 0.974, 0.921, 0.849, and 0.811, respectively. The sensitivity and specificity were high, and the results were similar to those of other studies. A recent study on cerebral spinal cord nerve sheath injury showed that NfL was a sign of axonal injury, and the concentration of NfL was related to the degree of axonal injury in an MRI (15). However, this study's authors did not evaluate the relationship between NfL and S100B concentrations and axonal injury, which had some limitations. Authors of follow-up studies maybe evaluate the degree of axonal injury and serum NfL and S100B concentrations by MRI, such as diffusion tensor imaging.

Overall, the level of serum NfL and S100B were positively correlated with the clinical prognosis of patients with aSAH. The detection of serum NfL and S100B levels helped in sensitively evaluating the severity of neuronal injury after aSAH. Serum NfL and S100B can be ideal biological markers for predicting aSAH. It was supportive in sensitively assessing the prognosis of patients, monitoring the development and curative effect of the disease, and improving the treatment methods in time, which is worthy of clinical attention.

## Data availability statement

The original contributions presented in the study are included in the article/supplementary material, further inquiries can be directed to the corresponding authors.

## Ethics statement

The studies involving human participants were reviewed and approved by the Dujiangyan Medical Center Ethics Committee, and written informed consent for participation was not required for this study in accordance with the national legislation and the institutional requirements.

## Author contributions

ZZ and QL conceived, designed the study, and wrote the report. YZha collected and compiled data. SY, YZha, YZho, and XY performed the statistical analysis and interpreted the data. All authors contributed to the article and approved the submitted version.

## Funding

This work was supported by the Sichuan Province Science and Technology Support Program (No. 140026) and Chengdu Medical Technology Project (No. 2020081).

## Conflict of interest

The authors declare that the research was conducted in the absence of any commercial or financial relationships that could be construed as a potential conflict of interest.

## Publisher's note

All claims expressed in this article are solely those of the authors and do not necessarily represent those of their affiliated organizations, or those of the publisher, the editors and the reviewers. Any product that may be evaluated in this article, or claim that may be made by its manufacturer, is not guaranteed or endorsed by the publisher.
